# Spatiotemporal Features of Retinal Waves Instruct the Wiring of the Visual Circuitry

**DOI:** 10.3389/fncir.2016.00054

**Published:** 2016-07-26

**Authors:** David A. Arroyo, Marla B. Feller

**Affiliations:** ^1^Department of Molecular and Cell Biology, University of California BerkeleyBerkeley, CA, USA; ^2^Helen Wills Neuroscience Institute, University of California BerkeleyBerkeley, CA, USA

**Keywords:** activity-dependent development, ferret, mouse, retinotopy, retinal waves, eye-specific segregation

## Abstract

Coordinated spontaneous activity is present in different sensory systems during early stages of development. This activity is thought to play a critical role in the development of sensory representations before the maturation of sensory experience. In the visual system, the mechanisms by which spatiotemporal properties of retinal spontaneous activity, called retinal waves, drive developmental events has been well studied. Recent advancements in pharmacological, genetic, and optogenetic manipulations have provided further understanding of the contribution of specific spatiotemporal properties of retinal waves to eye-specific segregation and retinotopic refinement of retinofugal projections. Here we review some of the recent progress in understanding the role of retinal waves in the early stages of visual system development, prior to the maturation of vision.

## Introduction

The cellular and molecular mechanisms underlying the spatiotemporal properties ofs retinal waves are well understood (Blankenship and Feller, 2010; Kerschensteiner, 2013). There are three developmental stages of waves mediated by different forms of neurotransmission; stage 1 waves are mediated by a combination of gap junctions and cholinergic circuits (Bansal et al., 2000), stage 2 waves are cholinergic (Feller et al., 1996), and stage 3 waves are glutamatergic (Wong et al., 2000). All three stages generate waves with different spatiotemporal properties (Maccione et al., 2014), as determined *in vitro*. Waves are characterized by their speed, frequency, covered area (size), within-burst spiking frequency, intra-retina distance-dependent correlations, and inter-eye correlations. More recently it has been established that the spatiotemporal properties of stage 2 retinal waves are preserved *in vivo* and are synaptically propagated to central visual targets (Colonnese and Khazipov, 2010; Ackman et al., 2012).

**Figure 1:**
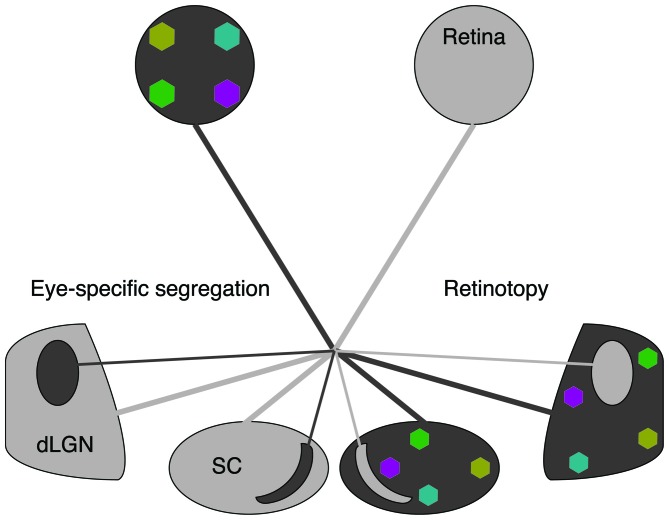
**Spontaneous retinal waves mediate eye-specific segregation and retinotopic refinement of retinofugal projections.** The axons of retinal ganglion cells (RGCs) target the dorsal lateral geniculate nucleus (dLGN) of the thalamus and the superior colliculus (SC). RGC projections from opposite eyes are segregated into ipsilateral and contralateral regions (black/gray oppositions). RGC projections from each retina are retinotopically organized (colored regions). Note that for simplicity we have depicted retinotopic maps only for one eye. Retinotopic maps and eye-specific segregations form for both eyes.

**Figure 2:**
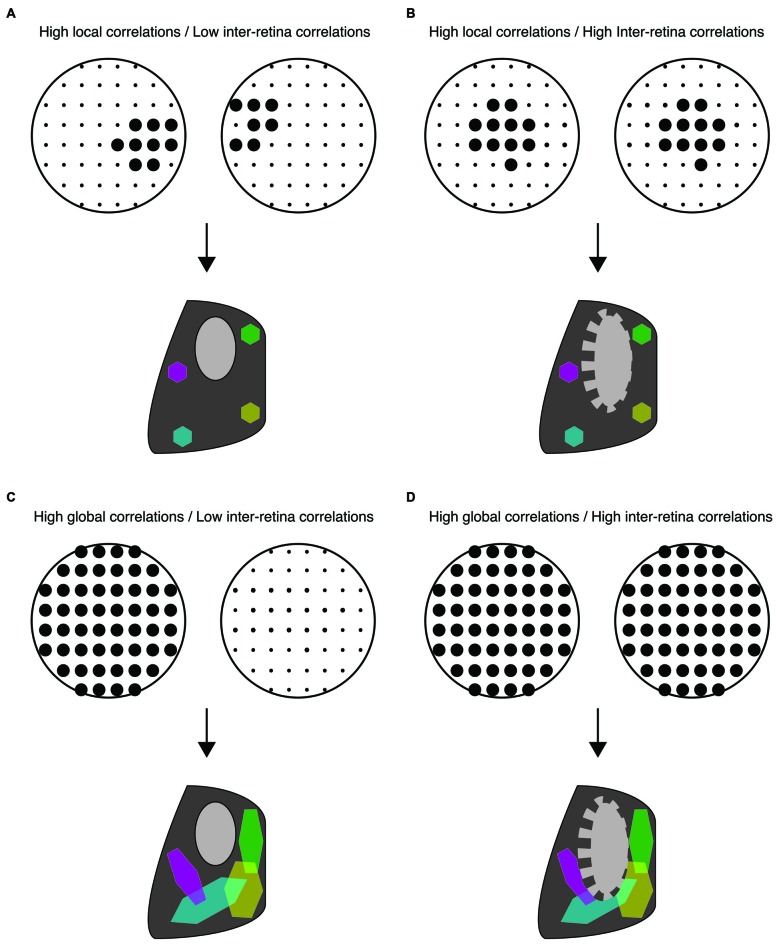
**Distinct spatiotemporal patterns of retinal waves instruct different features of retinofugal projections in mice. (A–D)** Top: schematic of retinal wave firing patterns. Dots are representative of RGCs with larger dots indicating elevated firing rates during a single retinal wave. Bottom: schematic of retinogeniculate wiring using same code as Figure 1—colored hexagons represent retinotopy while gray/black regions represent eye-specific segregation. **(A)** Under normal conditions, RGCs exhibit high local correlations while activity of the two retinas is minimally correlated. This pattern of activity supports both eye-specific segregation and retinotopic maps in both the dLGN and SC. **(B)** High correlations between retinal waves of opposite retinas induced by optogenetic stimulation is detrimental to eye-specific segregation while retinotopic maps are unaffected (Zhang et al., 2011). **(C)** Disruption of local correlations either by an increase in uncorrelated firing or by abnormally elevated correlations between distant RGCs (global correlations), such as that observed in β2KO and Retβ2-cKO mice, is detrimental to retinotopic map formation. Local correlations are sufficient for normal retinotopic maps in Retβ2-cKO, Rxβ2-cKO and β2(TG) (Xu et al., 2011, 2015; Burbridge et al., 2014). **(D)** High global correlations paired with high inter-retina correlations such as that observed in the β2KO mouse, is detrimental to both eye-specific segregation and retinotopic maps (Xu et al., 2011; Burbridge et al., 2014).

It is widely accepted that retinal waves are critical for the refinement of visual maps in retinofugal targets. Retinal projections to the superior colliculus (SC) and the dorsal lateral geniculate nucleus (dLGN) are retinotopically organized and segregate into eye-specific regions in a manner dependent on retinal activity (Figures 1, 2A; Wong, 1999; Bansal et al., 2000; Huberman et al., 2008). A classic debate in this field has been whether retinal waves are permissive or instructive for driving these developmental processes (Crair, 1999; Chalupa, 2009; Feller, 2009; Maccione et al., 2014). If waves are permissive, it implies that a minimum level of activity is required for some other instructive process to drive segregation, such as gene regulation. If waves are instructive, it implies that information about the target map is contained within the pattern of action potential firing during retinal waves. However, manipulations that alter the pattern of retinal waves also change their overall firing levels, making it difficult to differentiate between instructive and permissive roles. Here we review recent efforts that have used novel manipulations, in both mouse and ferret, to restrict perturbations of specific spatiotemporal properties of waves and thus advance the understanding of their contribution to the development of the visual circuitry.

## Inter-Eye Competition Instructs Eye-Specific Segregation

Inter-eye competition generated by spontaneous retinal waves was hypothesized to be instructive for eye-specific segregation of retinofugal projections (Butts et al., 2007). During retinal waves, individual retinal ganglion cells (RGCs) fire short bursts of action potentials that propagate to neighboring RGCs within a well defined region and are separated by 1–2 min of silence—hence the firing within retinotopically identified regions of the retina have stronger intra-retinal correlation than inter-retinal correlations. One of the unexpected findings of *in vivo* calcium imaging used to record activity of RGC terminals projecting to the SC was that activity of the two eyes is more correlated than would be expected by chance (Ackman et al., 2012). However, this correlation is weak and most waves occur independently.

The *β2KO* mouse is a classic model for studying the role of retinal waves in visual system development. This mouse model lacks the β2 subunit of the nicotinic acetylcholine receptor (β2-nAChR), which is required for cholinergic waves. This mouse has significantly impaired eye-specific segregation and retinotopic refinement of retinal projects to the dLGN of the thalamus and the SC. Unfortunately, there are several conflicting descriptions of β2KO retinal activity *in vit*ro, with different spatiotemporal properties reported based on recording conditions such as temperature and media used (McLaughlin et al., 2003; Sun et al., 2008; Kirkby and Feller, 2013). In contrast, sensitivity to temperature is not observed in waves of wild type (WT) mice (Stafford et al., 2009). Recent *in vivo* imaging of RGC axon terminals in the SC of *β2KO* revealed waves to be infrequent but dramatically larger and faster than waves in WT mice, and were described by the authors as “flashes” (Burbridge et al., 2014). Importantly, depolarizations induced by RGCs in *β2KO* mice were not sufficient to entrain postsynaptic SC neurons, which exhibited an activity pattern different from that of RGC axons. Hence *β2KO* have strong long-range intra-retinal correlations but weak and infrequent activity. Moreover, when flashes occur, they are likely to be correlated between the two eyes, indicating stronger inter-retinal correlations than those observed in WT (Burbridge et al., 2014).

Though these studies are consistent with the hypothesis that a lack of correlated activity between opposing retinas is important for eye-specific segregation (Figure 2B), they do not rule out the possibility that impaired map refinement in *β2KO* mice is due to an overall reduction in retinal activity. Recent studies have separated out the contributions of an overall decrease in retinal activity, the increase in inter-retinal correlations, and the disruption of the slowly propagating waves that drive local intra-retinal correlations. First, the frequency of waves is increased in the *β2KO* via intraocular injection of CP-cAMP (Burbridge et al., 2014), a manipulation known to increase the frequency of retinal waves *in vitro* (Stellwagen et al., 1999; Stellwagen and Shatz, 2002). Increasing wave frequency and thus overall wave activity improves eye-specific segregation in *β2KO*. Second, both correlated and anticorrelated inter-retinal activity were induced using optogenetic manipulations (Zhang et al., 2011). In these studies, correlated stimulation of the two retinas induced impaired eye-specific segregation while anticorrelated stimulation permitted eye-specific segregation and even partially rescued it in *β2KO* mice. These results were complemented by studies based on a transgenic mouse in which β2-nAChR subunits are knocked out exclusively from the retina starting early in development (*Rx-β2cKO* mice). In these mice local intra-retinal correlation properties of neighboring RGCs were largely maintained, but RGCs fired at low levels. This decrease in activity resulted in impaired eye specific segregation (Xu et al., 2015). Together, these studies strongly implicate that large-scale asynchronous activity between opposite eyes instructs eye-specific segregation while the local intra-retinal correlations induced by slowly propagating activity do not (Figures 2B,C).

Ferrets provide an interesting comparison to mice for assessing whether retinal waves are instructive for eye-specific segregation. Like mice, significant disruption of stage 2 cholinergic retinal waves disrupts eye-specific segregation of retinogeniculate afferents (Huberman et al., 2008). In contrast to mice, ipsilateral and contralateral retinal afferents of ferrets project to distinct cellular laminae in the dLGN, which may indicate differences in the mechanisms underlying visual map refinement between the two species. Using an immunotoxin to ablate starburst amacrine cells, a key component for generating Stage 2 retinal waves, Speer et al. (2014) were able to disrupt both local and global features of retinal waves while maintaining eye-specific segregation, similar to a previous study (Huberman et al., 2003). In contrast to mice, they found that large scale firing and the overall level of activity were not instructive, instead, the amount of uncorrelated firing exhibited by RGCs during the inter-wave periods was detrimental to eye-specific segregation.

A new insight into how waves influence eye-specific segregation in ferrets was achieved using an enucleated ferret model (Failor et al., 2015). Monocular enucleation results in expanded ipsilateral projections in the dLGN. Pharmacologically blocking waves in the remaining eye reduces this expansion and causes abnormal fragmentation of the ipsilateral laminae. To a lesser degree, blocking waves also induces fragmentation of contralateral projections in monocularly enucleated animals. This study suggests that intra-retinal activity itself plays a role in the development of eye-specific segregation that is independent of inter-eye competition. Furthermore, single-unit spike recordings from dLGN cells showed that blocking waves in monocularly enucleated animals causes abnormal enlargement and misalignment of receptive fields, thereby matching functional connectivity of the retino-geniculate projections with the observed anatomical phenotypes.

Together, these studies indicate that in ferrets intra-retinal correlations may be more critical for targeting axons to the correct layers and may also influence eye-specific segregation, in contrast with the observations in mice (Figures 2B,D).

## Local Intra-Retinal Correlations Instruct Retinotopy

In contrast to eye-specific segregation, local intra-retinal correlations induced by propagating activity are thought to be the primary feature instructing retinotopic refinement. During locally propagating activity, nearby RGCs are more correlated than distant RGCs and therefore information about retinotopy is contained within the retinal wave firing pattern (Eglen et al., 2003). The process of retinotopic refinement has been studied primarily in retinocollicular projections, though recent progress in understanding the role of waves in retinotopic refinement of retinogeniculate projections has also been made.

Several of the mouse models described above have also identified key features of retinal waves that instruct retinotopic refinement. For example, restoring the overall level of retinal activity in β2KO mice using optogenetics or intraocular injections of CP-cAMP rescued impairments in eye-specific segregation, but not retinotopy (Zhang et al., 2011; Burbridge et al., 2014; Figure 2D), suggesting that local intra-retinal correlations rather than overall retinal activity instruct retinotopy. One of the most striking mouse models lacks β2-nAChR in the nasal and temporal retina but expression is maintained along the central dorso-ventral strip (*Retβ2-cKO*), which serves as an internal control (Burbridge et al., 2014). *In vivo* calcium imaging of retinocollicular axon terminals confirmed that these mice exhibit locally propagating waves in the central retina while waves are absent in the nasal and temporal portions of the retina. Retinocollicular projections emerging from temporal or nasal retina were diffuse, indicating a lack of retinotopic refinement, while projections emerging from central retina were normal. This finding indicates that local intra-retinal activity patterns play a central role in establishing retinotopy (Figure 2C).

The *Rx-β2cKO* retina discussed earlier exhibits lower overall activity, smaller waves with high local correlations, more variable inter-wave intervals, and lower wave amplitude as measured with calcium imaging. *Rx-β2cKO* mice have disrupted eye-specific segregation and normal retinotopy in the monocular zones, indicating that the restricted local propagation of waves is sufficient for retinotopic refinement. Interestingly, retinotopy of the binocular zones was largely impaired, indicating interdependence between eye-specific segregation and retinotopy in the binocular zone (Xu et al., 2015). A careful comparison of waves spatiotemporal patterns in various knockout models is discussed in this study.

The role of waves in the retinotopic refinement of retinogeniculate projections in ferrets has also seen recent advancements. Intraocular blockade of inhibition to enhance glutamatergic stage 3 retinal waves led to abnormally early retinotopic refinement of retinogeniculate projections, as measured anatomically and functionally (Davis et al., 2015). Based on *in vitro* experiments, blockade of inhibition increases the frequency of glutamatergic waves while maintaining local correlations (Kerschensteiner and Wong, 2008; Firl et al., 2013). These findings are rather surprising since it suggests that increased spontaneous activity prior to visual experience could potentially compensate for lack of visual experience.

It is important to note that retinal waves work in concert with molecular cues for establishing retinotopic maps (Pfeiffenberger et al., 2006; Cang et al., 2008; Ackman and Crair, 2014; Assali et al., 2014). Though recent progress in this field is too vast to review here, we want to make note of a recent study that implicates a role for retinal waves in establishing heterogeneity in visual map organization not only across animals but also across the two colliculi of a single animal. The study makes a strong case that spontaneous activity and molecular cues interact stochastically to form well-defined yet variable retinotopic maps (Owens et al., 2015).

In summary, the evidence suggests that in mice, overall activity levels and inter-eye competition are determinant for eye-specific segregation while local intra-retinal correlations determine retinotopy, with some interdependence of the two in the binocular zones of central retinal targets. In ferrets, there is additional evidence that local intra-retinal correlations also influence eye-specific segregation.

## Emerging Roles of Retinal Waves in the Development of the Visual System

Most of the research on how retinal waves influence visual system development has been focused on retinofugal projections. However, there is also an interest in understanding whether retinal waves drive other aspects of visual system development. Recent studies have elucidated emerging roles for retinal waves in the development of visual circuits. In one study it was found that disrupting retinal waves increased cortical neurogenesis in the primary visual cortex while enhancing waves decreased neurogenesis (Bonetti and Surace, 2010). Another study found that eliminating the input of retinal waves to the SC resulted in migration and connectivity deficits in inhibitory interneurons of the dLGN (Golding et al., 2014). It has also been shown that elimination of retinal inputs to central targets accelerates the cortical innervation of the dLGN, yet synapse maturation was unaffected (Seabrook et al., 2013). In addition, computational models have implicated slow features of retinal waves in the establishment of complex cells in primary visual cortex (Dähne et al., 2014), and in the establishment of initial biases in orientation maps in primary visual cortex (Hagihara et al., 2015). These discoveries suggest a broad involvement of developmental spontaneous activity in cell signaling processes that mediate development, differentiation, integration, and connectivity of neural circuits.

The refinement of strategies to precisely control neural activity *in vivo*, as well as a deeper understanding of the molecular mechanisms linking retinal waves to various developmental processes will continue to provide insights onto the various roles of spontaneous activity patterns in circuit development.

## Author Contributions

Both authors contributed to the writing of the manuscript. All authors listed, have made substantial, direct and intellectual contribution to the work, and approved it for publication.

## Funding

This work was supported by the National Institutes of Health Grants R01EY013528 to MBF and Grant F31EY024842 to DAA.

## Conflict of Interest Statement

The authors declare that the research was conducted in the absence of any commercial or financial relationships that could be construed as a potential conflict of interest.
